# Surface electromyography signal processing and evaluation on respiratory muscles of critically ill patients: A systematic review

**DOI:** 10.1371/journal.pone.0284911

**Published:** 2023-04-27

**Authors:** Emanuel Fernandes Ferreira da Silva Junior, Shirley Lima Campos, Wagner Souza Leite, Pedro Vanderlei de Sousa Melo, Rômulo Aquino Coelho Lins, Maria das Graças Rodrigues de Araújo, Marcelo Renato Guerino

**Affiliations:** 1 Translational Health Graduate Program, Federal University of Pernambuco—UFPE, Recife, Pernambuco, Brazil; 2 Department of Physiotherapy, Federal University of Pernambuco—UFPE, Recife, Pernambuco, Brazil; 3 Health-applied Biology Graduate Program, Federal University of Pernambuco—UFPE, Pernambuco, Brazil; Complutense University of Madrid: Universidad Complutense de Madrid, SPAIN

## Abstract

**Background:**

Surface Electromyography (sEMG) has been used to monitor respiratory muscle function and contractility in several clinical situations, however there is the lack of standardization for the analysis and processing of the signals.

**Objective:**

To summarize the respiratory muscles most assessed by sEMG in the critical care setting and the assessment procedure details employed on those muscles regarding electrode placement, signal acquisition, and data analysis.

**Methods:**

A systematic review of observational studies was registered on PROSPERO (number CRD42022354469). The databases included PubMed; SCOPUS; CINAHL, Web of Science and ScienceDirect. Two independent reviewers ran the quality assessment of the studies using the Newcastle-Ottawa Scale and Downs & Black checklists.

**Results:**

A total of 311 participants were involved across the 16 studies, from which 62.5% (10) assessed the diaphragm muscle and 50% (8) assessed the parasternal muscle with similar electrode placement in both of them. We did not identify common patterns for the location of the electrodes in the sternocleidomastoid and anterior scalene muscles. 12/16 reported sample rate, 10/16 reported band-pass and 9/16 reported one method of cardiac-interference filtering technique. 15/16 reported Root Mean Square (RMS) or derivatives as sEMG-obtained variables. The main applicabilities were the description of muscle activation in different settings (6/16), testing of reliability and correlation to other respiratory muscles assessment techniques (7/16), and assessment of therapy response (3/16). They found sEMG feasible and useful for prognosis purposes (2/16), treatment guidance (6/16), reliable monitoring under stable conditions (3/16), and as a surrogate measure (5/16) in mechanically ventilated patients in elective or emergency invasive procedures (5/16) or in acute health conditions (11/16).

**Conclusions:**

The diaphragm and parasternal muscles were the main muscles studied in the critical care setting, and with similar electrodes placement. However, several different methods were observed for other muscles electrodes placement, sEMG signals acquisition and data analysis.

## 1. Introduction

Surface Electromyography (sEMG) signals have been recognized as a technological instrument for capturing respiratory myoelectric signals, and have been highlighted for the evaluation of respiratory neuromuscular function. In 2002, the American Thoracic Society/European Respiratory Society issued guidelines validating the use of sEMG signals as a method for analyzing respiratory neural triggering [1].

Respiratory muscle dysfunction is common in critically ill patients, and can lead to acute respiratory failure, also associated with increased hospital mortality, morbidity and hospital stay [2]. Therefore, it is crucial to assess respiratory functions, including the activity of the diaphragm and other respiratory muscles. Surface electromyography (sEMG) is a useful non-invasive asset that provides valuable information for monitoring the activity of these muscles [3] during breathing in critically ill patients.

sEMG signals can be employed to identify patients who are at risk for respiratory failure, monitoring the response to therapy, and guiding the timing of interventions [4,5]. Furthermore, they can offer insight into the patient’s level of effort during breathing [6], facilitate the assessment of the effectiveness of various ventilation strategies [6,7] and thus contribute to improving outcomes by allowing for earlier identification and intervention.

Despite the wide range of functionalities and benefits, the lack of standardization in sEMG can create issues, such as data inconsistency [8], due to non-standardized procedures for electrode placement, signal acquisition, and data analysis. This can lead to inaccurate diagnoses and ineffective treatments resulting from potential misinterpretation of data and respective errors. Moreover, limited comparability and replicability of sEMG protocols [9], which may hinder evidence-based practices and jeopardize progress in the field.

In 1999, the Surface EMG for Non-Invasive Assessment of Muscle (SENIAM) project aimed to integrate basic and applied research on sEMG for European cooperation. The project resulted in recommendations for sensor placement procedures and signal processing for 27 muscle segments, however, respiratory muscles were not included [10]. Thus, bringing the need for scientific evidence regarding the optimal sEMG procedures and analysis for respiratory muscles [11,12].

Based on the considerations presented, we conducted a systematic review of surface electromyography to address the following research questions:

1. What were the main respiratory muscles assessed through sEMG in the critical care setting?
2. What sEMG procedure details are described, regarding electrode placement, signal acquisition, and data analysis?

## 2. Methods

### 2.1 Design

This study was conducted as a systematic review, adhering to the PRISMA (2020) guidelines for reporting of systematic reviews and meta-Analysis [13] S4 Table. Details of the protocol for this review were registered on PROSPERO (number CRD42022354469).

### 2.2 Data sources and search strategy

Five databases were searched for this review, including Medical Literature Analysis and Retrieval System Online (Medline) of the United States National Library of Medicine (PubMed), SCOPUS, Cumulative Index to Nursing and Allied Health Literature (CINAHL), Web of Science and ScienceDirect. The bibliographic survey was conducted until August 11, 2022 with no restrictions on language or year of publication. The search terms were formulated using adaptation to the PICO framework, and indexed terms from the Medical Subject Headings (MeSH) metadata system were combined with and keywords, “surface electromyography”; “respiratory muscles” and “intensive care unit”, to create a comprehensive search strategy tailored to each database’s particularities, as described in S1 Appendix.

### 2.3 Eligibility criteria

The inclusion criteria for this study were as follows: (1) study design—observational studies, including retrospective, prospective cohort, case-control, and cross-sectional; (2) assessment instrument—respiratory assessment using sEMG; (3) population–adults aged 18 years or older; (4) Condition: admitted to an intensive care unit. Studies that applied other methods of muscle electrical activity assessment aside from sEMG, such as needle EMG, were excluded, as well as those focusing solely on burn patients or patients requiring neuromuscular blockers, or missing information of sEMG parameter configuration or signal processing.

### 2.4 Screening and data extraction

**Fig 1:**
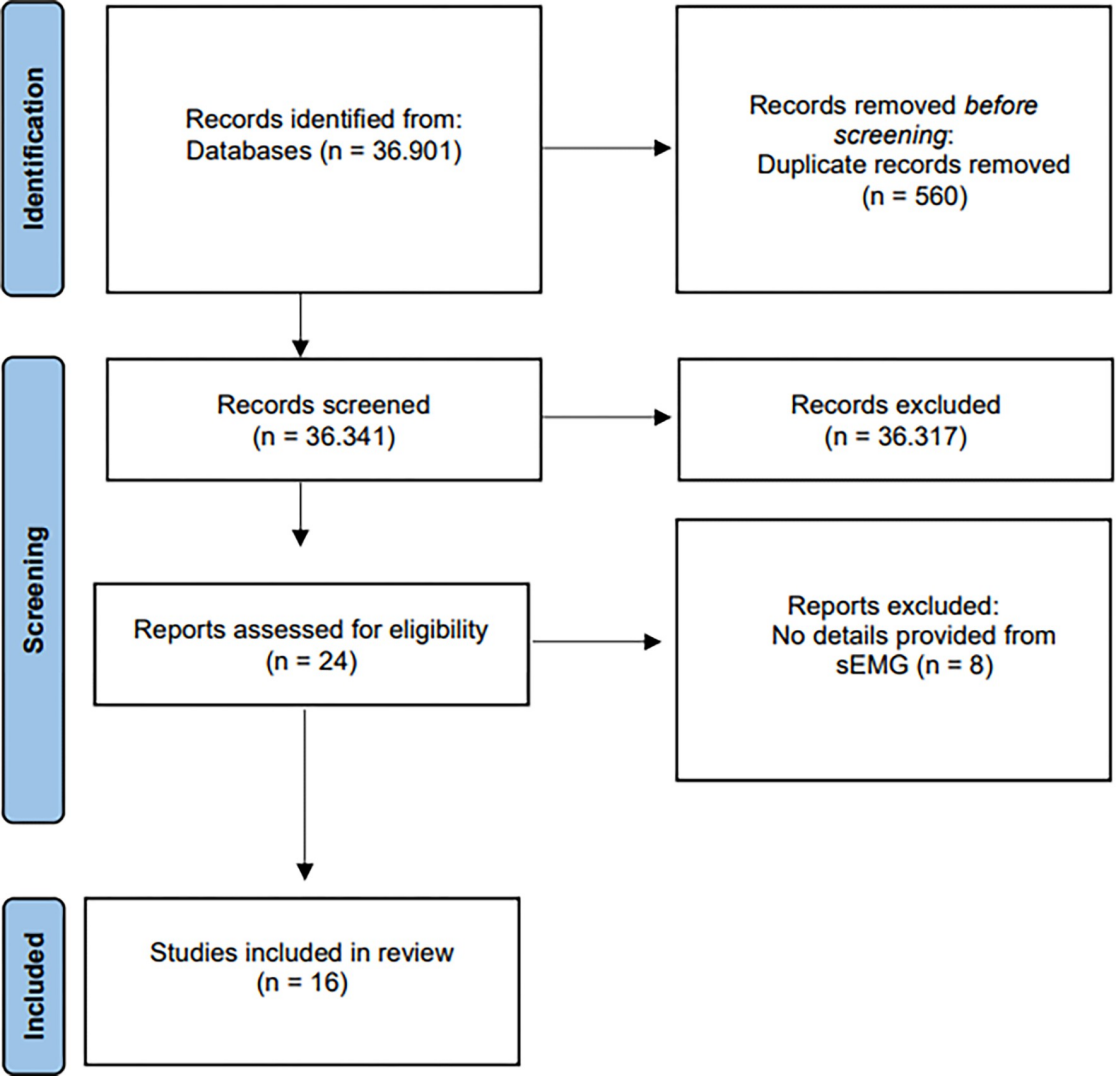
Identification of studies through databases according to Preferred Reporting Items for Systematic Reviews and Meta-analyses (PRISMA).

First, the eligible studies had their title and abstract screened using the Systematic Review-Rayyan [14] synthesis software by two independent reviewers (E. F. F. S. J. and W. S. L.). Secondly, full-text screening was performed on the selected studies, and in cases of disagreement between the pair of reviewers, a third assessor was consulted to resolve discrepancies. Finally, the data extracted comprised: author, year, country, study objectives, study design, sample characteristics, and sEMG data-related variables, such as respiratory muscles assessed, electrode placement, signal acquisition, and data analysis. The flowchart of included and excluded localized literature is represented using terms described in (Fig 1).

### 2.5 Quality assessment

The quality appraisal of observational studies was conducted using two checklists: Newcastle-Ottawa [15] and Downs & Black scales [16]. The Newcastle-Ottawa scale comprises three domains: selection, comparability, and exposure receiving scores from zero to nine. The resulting interval scores are used to qualify the studies into Good (7–9 points), Satisfactory (5–6 points), or Poor (0–4 points) quality [15]. The scale presents a slight difference in the scoring system for case-control and cross-sectional studies.

The Downs & Black scale comprises a questionnaire consisting of 27 items categorized into five domains: reporting (10 items), external validity (3 items), study bias (7 items), confounding factors (6 items), and study power (1 item). The final items are scored as yes or no responses. The final scores assigned to studies are used to determine their methodological quality with scores categorized as: Excellent (26–28); Good (20–25); Righteous (15–19); and Poor (≤14) points [16].

## 3. Results

The total of 36,901 articles were initially screened, then reduced to 36,341 after excluding 560 duplicates. An additional 36,317 articles were excluded based on their failure to meet the inclusion criteria after the title/abstract screening and further 8 articles were excluded due to missing information of sEMG parameter configuration or signal processing. As a result of this screening process, 16 articles were included in this review (Fig 1).

### 3.1 Quality assessment

15 studies (93.75%) were scored as cross-sectional design and one study (6.25%) were scored as case-control design. By using the Newcastle-Ottawa scale, 11 studies (68.75%) were rated as having “satisfactory” quality, while five studies (31.25%) were classified as “good” Table 1. None of the studies were considered to be of poor methodological quality, as detailed in S1 and S2 Tables. Meanwhile, using the Downs & Black scale, 13 studies (81.25%) were classified as having fair methodological quality, while three studies (18.75%) were considered to be of “poor” quality, as outlined in S3 Table.

**Table 1:** Quality assessment of the included studies.

Author, year, Country	Study design	Methodological quality (Score)	
		Newcastle-Ottawa Scale	Downs & black Scale
Pozzi et al. (2022) [17], Italy	Cross-sectional	6 (Satisfactory)	15 (Fair)
Graßhoff et al. (2021) [18], Germany	Cross-sectional	6 (Satisfactory)	15 (Fair)
Bureau et al. (2021) [19], France	Cross-sectional	7 (Good)	15 (Fair)
Lokin et al. (2020) [20], Netherlands	Cross-sectional	7 (Good)	15 (Fair)
Roesthuis et al. (2020) [21], Netherlands	Cross-sectional	7 (Good)	16 (Fair)
Souza Costa et al. (2020) [22], Brazil	Cross-sectional	7 (Good)	15 (Fair)
Bellani et al. (2018) [6], Italy	Cross-sectional	5 (Satisfactory)	15 (Fair)
Duarte et al. (2017) [23], Brazil	Cross-sectional	5 (Satisfactory)	15 (Fair)
Sánchez et al. (2017) [24], Colombia	Case-control	7 (Good)	15 (Fair)
Ortega et al. (2017) [25], Colombia	Cross-sectional	6 (Satisfactory)	15 (Fair)
Walterspache et al. (2017) [26], Germany	Cross-sectional	6 (Satisfactory)	16 (Fair)
Cecchini et al. 2014 [27], France	Cross-sectional	6 (Satisfactory)	15 (Fair)
Schmidt et al. 2013 [28], France	Cross-sectional	5 (Satisfactory)	17 (Fair)
Tassaux et al. 2005 [29], Switzerland	Cross-sectional	5 (Satisfactory)	14 (Poor)
Tassaux et al. (2002) [30], Switzerland	Cross-sectional	6 (Satisfactory)	14 (Poor)
Imsand et al. 1994 [31], Switzerland	Cross-sectional	5 (Satisfactory)	12 (Poor)

### 3.2 Outcome: sEMG processing variables for respiratory muscles

Table 2 shows the processing data for the sEMG analysis. Sampling rate was mentioned in twelve studies (75%) obtaining variations between 500 and 10,000 Hz [6,17–19,21,23–27,29,30], Bandpass filters were reported in 10 studies (62.5%) with variations between 0–1,000 Hz [6,17,19,21,22,24–27,31]. Only one study (6.25%) reported high-pass and low-pass filters for EMG signal processing details [26].

**Table 2:** Summary of respiratory muscle sEMG processing variables.

Author/Year	Respiratory muscle sEMG processing data									
	Sample rate (Hz)	LPF (Hz)	HPF (Hz)	Band-pass (Hz)	CIF technique	Amplifier Gain	Data acquisition duration	Unit	Measures	Software
Pozzi et al. (2022) [17]	500	NR	NR	0–200	Gating	NR	180s	μV	RMS	LabChat
Graßhoff et al. (2021) [18]	1.000	NR	NR	NR	Gating	NR	NR	μV	RMS	NR
Bureau et al. (2021) [19]	10.000	NR	NR	40–500	NR	NR	600s	NR	EMG_MAX_/ EMG_AUC_	LabChart
Lokin et al. (2020) [20]	NR	NR	NR	NR	Gating	NR	900s	μV	RMS	MATLAB R2016a
Roesthuis et al. (2020) [21]	2.048	NR	NR	25–500	Wavelet-based adaptive filter	NR	300s	%	EMG_PEAK_; EMG_AUC/min_	MATLAB R2014b
Souza Costa et al. (2020) [22]	NR	NR	NR	20–500	NR	NR	60s	μV	RMS	NR
Bellani et al. (2018) [6]	500	NR	NR	0–200	Gating	20	NR	μV	RMS	Dipha Software
Duarte et al. (2017) [23]	500	NR	NR	NR	NR	NR	NR	NR	RMS	NR
Sánchez et al. (2017) [24]	1.024	NR	NR	20–500	NR	NR	1.200s	NR	RMS	NR
Ortega et al. (2017) [25]	1.024	NR	NR	10–500	RLS adaptive 5^th^-order filter	NR	180s	NR	NR	NR
Walterspacher et al. (2017) [26]	10.000	500	1.000	20–1.000	Visual inspection	1000	120s	%	RMS	LabChat v.7.2
Cecchini et al. (2014) [27]	2.000	NR	NR	10–1.000	NR	500	180s	%	RMS	NR
Schmidt et al. (2013) [28]	NR	NR	NR	NR	NR	NR	NR	NR	EMG_MAX_; EMG_AUC_	NR
Tassaux et al. (2005) [29]	1.000	NR	NR	NR	NR	NR	60s	NR	EMG signal	NR
Tassaux et al. (2002) [30]	2.000	NR	NR	NR	50-Hz filter	NR	600s	NR	EMG_AUC_	AcqKnowledge
Imsand et al. (1994) [31]	NR	NR	NR	15–1.000	Manual gating	NR	60s	%	EMG signals	NR

LPF: Low-pass filter; HPF: High-pass filter; CIF: Cardiac-interference filtering; NR: Not reported; RMS: Root mean square; EMG: Electromyogram; EMG_AUC_: Area under the EMG signal curve; EMG_max_: The difference between the maximum amplitude of RMS signal and its baseline; EMG_peak_: Peak of muscle activity during inspiration; EMG_AUC/min_: End of muscle activity per breath.

Regarding cardiac-interference filtering techniques to prevent EMG noise contamination, nine studies (50%) have reported a number of techniques, including gating [6,17,18,20,31], or some sort of adaptive filter [21,25,30] or even visual inspection [26]. Amplifier gain is one of the settings for high quality EMG signals with low noise. This setting is defined as the ratio between the voltage that enters and exits the amplifier, should suit the characteristics of the experiment, the type of electrodes used, as well as the muscle studied, and not the adjusted to exceed the voltage expected from the system [32]. Gain was mentioned in three studies (18.75%) with variations between 20–1,000 times [6,26,27]. EMG data acquisition recording reported by twelve studies (75%) varied from 60 through 1.200 seconds [17,19–22,24–27,29–31] Table 2.

Regarding the sEMG signal units, five papers (31.25%) [6,17,18,20,22] presented them in microvolts (μV), four studies (25%) [21,26,27,31] in percentages, and eight studies (50%) did not inform any unit [19,23–25,28–31]. The obtained variables were presented as simple mean RMS/EMG signal usually rectified or normalized [6,17–20,22–24,26,27,29] or integrated RMS/EMG signals—the difference between the maximum amplitude of RMS signal and its baseline (EMG_MAX_), area under the EMG signal curve (EMG_AUC_), peak of muscle activity during inspiration (EMG_PEAK_), and end of muscle activity per breath (EMG_AUC/min_) [19,21,28,30,31] Table 2.

For software dedicated to sEMG signal offline analysis, only seven (43.75%) reported which one they used. Labchart was the most reported by three studies (18.75%) [17,19,26], followed by two reporting Matlab (12.5%) [20,21] and two reporting Dinha (6.25%) [6], and Acqknowledge (6.25%) [30] Table 2.

### 3.3 Outcomes: Characteristics of the studies, technical parameters of sEMG of the respiratory muscles and clinical applicability

A total of 311 participants were involved across the 16 studies, with 15 studies (66.2%) reporting male participants as the majority. The average age of the participants ranged from 62.1 ± 10.56 years Table 3.

**Table 3:** Summary of sample characteristics, technical parameters of sEMG of the respiratory muscles, clinical use and respective studies’ findings.

Author, year, Country	Objective	Sample characteristics	Body position, skin electrode placement and muscles assessed	Main findings
Pozzi et al. (2022) [17], Italy	To describe respiratory muscle activation by sEMG during an spontaneous breathing trial and to assess their electrical activity in successful vs. failing weaning patients.	*N*. *of participants*: 37. *Age*: 56 (50–62). *Sex*: M: 27/ F: 10. *Condition*: COPD, Diabetes, ARDS, atrial fibrillation, Septic shock. *Inclusion criteria*: Patients who were on IMV for > 48 h eligible for spontaneous breathing trial.	**Body position:** Not reported. **Reference electrode**: Not reported. **Inter electrode distance**: Not reported. **Diaphragm**: Lower costal margin, bilaterally on the midclavicular line. **Parasternals**: 2nd intercostal space, bilaterally in the parasternal line. **Sternocleidomastoid**: In the middle third of the sternocleidomastoid muscle, on the right or left side (usually opposite the central venous line). **Rectus abdominis:** On the left midclavicular line, at the level of the umbilicus.	Respiratory muscles’ electrical activity increased during spontaneous breathing trial. Recruitment of expiratory muscles quantified by sEMG is associated with this trial failure.
Graßhoff et al. (2021) [18], Germany	To investigate the estimation of the pressure-time product of inspiratory muscle pressure via sEMG by identifying a patient-specific conversion factor during end-expiratory occlusions.	*N*. *of participants*: 43. *Age*: 64 ± 11. *Sex*: M: 34/ F: 9. *Condition*: Patients scheduled for elective bronchoscopy. *Inclusion criteria*: Intubated, moderate/deep sedation level, on assisted spontaneous ventilation.	**Body position:** Not reported. **Reference electrode:** Sternum. **Inter electrode distance**: Not reported. **Diaphragm**: Bilaterally at the lower costal margin on the midclavicular line. **Parasternals**: Bilaterally in the 2° intercostal space on the parasternal line.	sEMG was well correlated with pressure-time product. sEMG can be used as a non-invasive alternative for monitoring the patients’ inspiratory effort under assisted mechanical ventilation.
Bureau et al. (2021) [19], France	To evaluate the respective impact of customized PAV and PSV settings on patient-ventilator asynchrony.	*N*. *of participants*: 34. *Age*: 66 (57–77). *Sex*: M: 25 / F: 9. *Condition*: Patients on PSV under IMV for > 24 h, for respiratory cause with hypoxemia. *Inclusion criteria*: Sedated in IMV.	**Body position:** Not reported. **Reference electrode**: Not reported. **Inter electrode distance:** 2 cm. **Parasternals**: 2° intercostal space lateral to the estrum. **Alae nasi muscles**: Placed on the lateral surfaces of the nose.	Parasternal intercostal muscles electrical activity and asynchrony index were lower with PAV and other PSV modes. Mechanical ventilation modes did not decrease dyspnea and activity of muscles, even though PAV was able to reduce asynchrony more effectively.
Lokin et al. (2020) [20], Netherlands	To compare the ability of diaphragm activity detection between surface electromyography of the diaphragm and transesophageal electromyography of the diaphragm.	*N*. *of participants*: 15. *Age*: 62 (45–72). *Sex*: M: 9 / F: 6. *Condition*: Adults with clinical complications, elective and emergency surgery. *Inclusion criteria*: At least 48 h of IMV in PSV.	**Body position:** Not reported. **Reference electrode**: Sternum. **Inter electrode distance**: Not reported. **Diaphragm**: Bilaterally below the front and lower dorsal ribs.	sEMG was not reliable for breathing effort detection in subjects who were invasively ventilated compared with transesophageal electromyography. In stable recordings, however, both of the methods had excellent temporal correlation and good agreement.
Roesthuis et al. (2020) [21], Netherlands	To investigate the effect of different inspiratory support levels on the recruitment pattern of extradiaphragmatic inspiratory muscles and to evaluate agreement between activity of extradiaphragmatic inspiratory muscles and the diaphragm.	*N*. *of participants*: 17. *Age*: 64 ± 8.2. *Sex*: M: 12 / F: 5. *Condition*: COPD, pneumonia, ARDS, abdominal aortic aneurysm. *Inclusion criteria*: At least 3 days in IMV, light/some deep sedation.	**Body position:** Not reported. **Reference electrode** Patient’s pulse. **Inter electrode distance**: Not reported. **Scalenes/Sternocleidomastoid**: Lower third of the muscles. **Parasternals**: 2nd intercostal space, 3 cm lateral to the sternum. **Alae nasi muscles**: Placement of an electrode in each nostril **Genioglossus**: Below the chin and above the hyoid bone.	Extradiaphragmatic inspiratory muscle activity increases in response to lower inspiratory support levels. However, there is a poor correlation and agreement with the change in diaphragm activity.
Souza Costa et al. (2020) [22], Brazil	To analyze the respiratory muscle groups involved with the timed inspiratory index index utilizing the sEMG.	*N*. *of participants*: 46. *Age*: 80 (71–87). *Sex*: M: 26 / F: 20. *Condition*: Sepsis, acute respiratory failure, stroke and heart failure. *Inclusion criteria*: Patients who were on IMV for > 24 h, clinically ready for the weaning process.	**Body position:** 45°. **Reference electrode**: Not reported. **Inter electrode distance**: Not reported. **Diaphragm**: In the lower intercostal spaces on the right side of the body, in the midclavicular line. **Scalenes**: Not reported. **Sternocleidomastoid**: Not reported. **External intercostals**: In the 5th intercostal space on the posterior axillary line.	Subjects succeeding in a weaning trial had higher muscle strength. A vigorous diaphragm with low fatigue potential seems essential for successful weaning.
Bellani et al. (2018) [6], Italy	To assess the reliability of sEMG of the respiratory muscles for monitoring diaphragm electrical activity and subject effort during assisted ventilation.	*N*. *of participants*: 14. *Age*: 56 ± 12. *Sex*: M: 12 / F:2. *Condition*: Pneumonia, ARDS, sepsis, trauma. *Inclusion criteria*: Patients intubated or tracheostomized in PSV mode.	**Body position:** Not reported. **Reference electrode**: Not reported. **Inter electrode distance**: Not reported. **Sternocleidomastoid:** Middle third of the SCM muscle. **External intercostals**: 2th intercostal space, bilaterally on the parasternal line, for parasternal external intercostal muscles. **Diaphragm**: Inferior costal margin, bilaterally in the midclavicular line, for costal diaphragm.	Diaphragm activity can be reliably monitored by both electrical activity measured by nasogastric tube with electrodes and sEMG.
Duarte et al. (2017) [23], Brazil	To evaluate the diaphragmatic surface electromyography of postoperative liver transplantation subjects.	*N*. *of participants*: 14. *Age*: 57 ± 7.1. *Sex*: M: 10 / F:4. *Condition*: Liver transplantation. *Inclusion criteria*: Patients eligible for extubation in PSV.	**Body position**: 35°. **Reference electrode**: Not reported. **Inter electrode distance:** 16 cm. **Diaphragm**: Two electrodes in the paraxiphoid region, 5 cm below the xiphoid process, and the other 2, in the region of the bilateral costal margin, with approximately 16 cm between them.	There were significant differences between the root mean square of the diaphragm domes under mechanical ventilation and after extubation, showing lower effectiveness of the diaphragm muscle against resistance.
Sánchez et al. (2017) [24], Colombia	To assess the engagement of the respiratory muscles as an index during spontaneous breathing trial and to associate the level of engagement with the muscular weakness in poisoned patients.	*Case group* *N*. *of participants*: 23. *Age*: 30.3 ± 10. *Sex*: M: 14 / F:9. *Condition*: Patients poisoned by organophosphate. *Inclusion criteria*: Patients who were on IMV during spontaneous breathing trial. *Control group* *N*. *of participants*: 28. *Age*: 27 ± 6.5. *Sex*: M: 21/ F:7. *Condition*: Healthy patients. *Inclusion criteria*: Received non-invasive ventilation for 900 second.	**Body Position**: Not reported. **Reference electrode**: Not reported. **Inter electrode distance**: Not reported. **Diaphragm**: Anterolateral portion of the thorax, between the 6th and 8th intercostal space, between the mid-axillary line and the external clavicular line **Sternocleidomastoid**: 20% of the distance between the mastoid apophysis and the sternal notch.	Engagement of respiratory muscles index is a promising index to assess the level of participation of respiratory muscle on spontaneous breathing test in patients poisoned, which could improve the extubation prognosis for these patients.
Ortega et al. (2017) [25], Colombia	To evaluate the feasibility of sEMG derived indexes in predicting weaning outcomes among mechanically ventilated subjects after cardiac surgery.	*N*. *of participants*: 10. *Age*: 63.14 ± 16.9. *Sex*: M: 10. *Condition*: Postoperative cardiac surgery. *Inclusion criteria*: Clinically stable ready for IMV weaning.	**Body position**: Not reported. **Reference electrode**: Not reported. **Inter electrode distance**: Not reported. **Diaphragm**: Between the 7th and 8th intercostal spaces between the midaxillary line and the external clavicular line.	The obtained indexes show a correlation between high expenditure and weaning test failure. sEMG is becoming a promising procedure for assessing the state of mechanically ventilated patients.
Walterspache et al. (2017) [26], Germany	To assess the acute effects on the neural respiratory drive measured by sEMG and the diaphragm in different body positions during mechanical ventilation.	*N*. *of participants*: 9. *Age*: 66 ± 18. *Sex*: M: 6 / F: 3. *Condition*: Lung transplantation and stroke. *Inclusion criteria*: Tracheostomized patients hemodynamically stable in spontaneous breathing trial.	**Body position**: Supine (lying down), semi-sitting at 30° and sitting at 80° in bed. **Reference electrode**: Not reported. **Inter electrode distance**: 2cm. **Diaphragm**: Bilaterally on the lower costal margin, 16 cm lateral to the common electrode, placed 5 cm cranial to the xiphoid. **Parasternals**: Bilaterally in the 2nd intercostal space.	Diaphragm’s electrical activity changed under spontaneous breathing from supine to sitting and between semirecumbent to sitting. Body positioning influences respiratory drive to the diaphragm in tracheotomized patients during unassisted breathing.
Cecchini et al. 2014 [27], France	To compare electrical activity of diaphragm, scalene, and alae nasi, according to the ventilatory mode and assist level.	*N*. *of participants*: 12. *Age*: 68 ± 8. *Sex*: Not reported. *Condition*: Patients with acute respiratory failure, pneumonia, ARDS, pulmonary edema, septic and cardiogenic shock *Inclusion criteria*: Adults in IMV capable of triggering PSV/NAVA.	**Body position**: Not reported. **Reference electrode**: Not reported. **Inter electrode distance**: Not reported. **Scalenes**: In the posterior triangle of the neck, at the level of the cricoid cartilage. **Alae nasi muscles**: They were obtained by placing an electrode in each nostril.	NAVA and PSV both reduced extradiaphragmatic inspiratory muscle activity, in proportion to the level of assistance. NAVA resulted in a predominant contribution of the diaphragm to inspiratory effort.
Schmidt et al. 2013 [28], France	To determine if sEMG of extradiaphragmatic inspiratory muscles vary with PSV settings and relate to the degree of discomfort and the intensity of dyspnea in acutely ill patients.	*N*. *of participants*: 12. *Age*: 60 ± 15. *Sex*: M: 9 / F: 3. *Condition*: Patients with pneumonia, COPD and septic shock. *Inclusion criteria*: Patients intubated or tracheostomized in IMV weaning process.	**Body position**: Not reported. **Reference electrode**: Not reported. **Inter electrode distance**: Not reported. **Scalenes**: Bilateral registers, positioned in the posterior triangle of the neck at the level of the cricoid cartilage, between the sternocleidomastoid muscle and the clavicle. **Parasternals**: Bilateral records, positioned in the 2nd intercostal space, close to the sternum. **Alae nasi muscles**: Placement of an electrode in each nostril.	sEMG of extradiaphragmatic inspiratory muscles provides a reliable and non-invasive indicator of respiratory muscle loading/unloading in mechanically ventilated patients. It is strongly correlated to the intensity of dyspnea, and could be used as a surrogate of respiratory sensations.
Tassaux et al. 2005 [29], Switzerlan	To test the hypothesis that increasing the expiratory trigger set-point could reduce the magnitude of intrinsic PEEP, the number of non triggering breaths, the level of inspiratory effort and triggering delay.	*N*. *of participants*: 10. *Age*: 68.7 ± 10. *Sex*: M: 7 / F: 3. *Condition*: Patients with acute respiratory failure with COPD *Inclusion criteria*: Intubated patients in IMV weaning phase.	**Body position**: Not reported. **Reference electrode**: Sternum. **Inter electrode distance**: Not reported. **Diaphragm**: Bilaterally on the costal margin.	Setting expiratory trigger at a higher percentage of peak inspiratory flow in patients with obstructive disease during pressure support improves patient–ventilator synchrony and reduces inspiratory muscle effort.
Tassaux et al. (2002) [30], Switzerlan	To compare the effects of ASV and SIMV-PS modes on patient-ventilator interactions in mechanical ventilation.	*N*. *of participants*: 10. *Age*: 71 ± 9.6. *Sex*: F: 6 / M: 4. *Condition*: Patients with COPD exacerbation, acute pneumonia, asthma and sepsis. *Inclusion criteria*: Intubated patients in weaning procedure by SIMV-PS or ASV mode.	**Body position**: Not reported. **Reference electrode**: Not reported. **Inter electrode distance**: Not reported. **Sternocleidomastoid**: Placed over the muscle (palpation) and the mastoid process.	ASV provided levels of minute ventilation comparable to those of SIMV-PS. Central respiratory drive and sternocleidomastoid activity were markedly reduced in ASV, suggesting decreased inspiratory load and improved patient-ventilator interactions.
Imsand et al. 1994 [31], Switzerland	To assess quantitatively the effect of varying the level of machine assistance on the electrical activity amplitude of inspiratory muscles.	*N*. *of participants*: 5. *Age*: 58.6 ± 8. *Sex*: M: 1 / F: 4. *Condition*: COPD *Inclusion criteria*: intubated patients, hemodinamically stable, conscious and not sedated.	**Body position**: 45°. **Reference electrode**: Not reported. **Inter electrode distance**: Not reported. **Sternocleidomastoid**: Muscle body.	The durations of electrical activation and the integrated electromyograms of diaphragm and sternocleidomastoid were similar in successive spontaneous and assisted breaths, neither changed with increasing level of machine assistance.

ARDS: Acute respiratory distress syndrome; cm: Centimeters; COPD: Chronic obstructive pulmonary disease; sEMG: Surface electromyography; ICU: Intensive care unit; IMV: Invasive mechanical ventilation; PEEP: Positive end-expiratory pressure; NAVA: Neurally adjusted ventilatory assist; PAV: Proportional assist ventilation; PSV: Pressure support ventilation; ASV: Adaptive support ventilation; SIMV-PS: Synchronized intermittent mandatory ventilation plus pressure support, F: Female; M: Male; mm: Millimeters; RMS: Root mean square.

According to the objectives of the included studies, the applicability of sEMG used in mechanically ventilated patients can be summarized to the following categories, description muscles activation in different settings [17,21–23,28,31], testing of reliability and correlation to other respiratory muscles assessment techniques [6,18,20,24–27] and assessment of therapy response [19,29,30]. These studies have found that sEMG was feasible and resourceful tool for prognosis purposes [17,22] treatment guidance [19,26,27,29–31], reliable monitoring under stable conditions [20,23,28] and as surrogate measure [6,18,21,24,25] Table 3.

The mechanically ventilated patients’ scenarios for sEMG assessment employment were either in the elective or emergency invasive procedures [18,20,23,25,26] or in acute health conditions [6,17,19,21,22,24,27–30] Table 3.

Scalene muscles (ESC) were analyzed in four studies (25%) [21,22,27,28] however, information related to electrode placement was described in only three studies (18.75%), guided by ultrasound positioned in the lower region of the muscle third [21], positioned in the posterior triangle of the neck, at the level of the cricoid cartilage [27] and in bilateral recordings, positioned in the posterior triangle of the neck at the level of the cricoid cartilage, between the sternocleidomastoid muscle and the clavicle [28]. Some studies did not provide sufficient information to determine placement [22] Table 3.

Sternocleidomastoid (SCM) muscle analysis was performed in seven studies (43.7%) [6,17,21,22,24,30,31], therefore, information related to electrode placement was described in only six studies (37.5%) rejecting each other, being its placement in the middle third, identified by palpation on the right or left side, generally opposite to the medications due to the fixation of the central venous catheter [6,17], guided by ultrasound in the lower third of the muscle [21], 20% of the distance between the mastoid apophysis and the sternal furcula [39], positioned over the muscle “palpation” and the mastoid process [30] and fixed on the muscle body [31]. The other studies did not provide information to determine the placement [22] Table 3.

The terminology referring to the parasternal has been characterized by the intercostal portion parallel to the sternum, however, it may be linked to the intercostal muscles, referring to the fibers parallel to the sternum [33–35]. The selected papers described and analyzed the parasternal muscles in eight studies (50%), under the positioning in the second intercostal space [6,17–19,21,26,28], and electrodes placed in the fifth intercostal space in the posterior axillary line2, however only [6,17,18,26] placed the electrodes bilaterally Table 3.

The positioning of the electrodes on the Diaphragm (DIA) was measured in ten studies (62.5%), attached to the lower intercostal spaces in the midclavicular line [6,17,18,20,22,24,25,29] that of these only three studies analyzed the right hemibody [22,24,25] and positioning two electrodes in the paraxiphoid region, 5 cm below the xiphoid process, and the other 2, in the bilateral costal margin region, with a distance of approximately 16 cm between them [23,26]. Only one study (6.25%) reported analyzing the Rectus Abdominal muscle with its positioning on the left midclavicular line, at the level of the umbilicus [17] Table 3.

The alae nasi muscles were analyzed in only four studies (25%) [19,21,27,28] with its electrodes for electromyographic capture placed in each nostril. The genioglossus muscle was analyzed in only one study (6.25%), reliably reporting its positioning, characterized below the chin and above the hyoid bone [21]. In relation to the reference electrode, only four studies (25%) reported its placement, three studies (18.75%) under the sternum [18,20,29], and one study (6.25%) attached to the patient’s wrist [21].

The interelectrode distance affects the signal quality and determines the volume of muscle fibers that will be measured, therefore, affecting the selectivity of recording. This parameter was reported in three studies (18.75%) which reported its distance of 2 cm [19,26] and 16 cm [23]. Four studies (25%) reported the angle positioning of patients: 35° [19], 45° [22,31] and in three different body positions, supine at 0°, semirecumbent at 30° and sitting at 80° on the bed [26] Table 3.

## 4. Discussion

This review presents findings indicating that the diaphragm and parasternal muscles were the most commonly used for assessing respiratory function through sEMG in critically ill patients admitted to the ICU. To the best of our knowledge, this is the first systematic review to comprehensively summarize the methodological aspects of the evaluation protocols and sEMG signals processing of respiratory muscles in patients admitted to the ICU.

Upon analyzing the respiratory electromyographic data processing and analysis procedures in the included studies, it was found a lack of standardization in the target respiratory muscles, electrode placement and data acquisition details. Therefore, the studies could not be subjected to quantitative analyses or meta-analyses. A wide range of significant variation in the anatomical sites for skin electrode placement, especially for muscles underlying other larger muscles. This could be minimized by implementing other resources, such as ultrasound guidance, as demonstrated by Roesthuis et al. [21], although Imsand et al. [31] presented some respiratory muscles feasible to be tracked for electrode placement by palpating muscle body.

The diaphragm and parasternal muscles were the most common for sEMG assessment in critically ill patients in the ICU, likely due to their crucial role in respiratory function. The diaphragm electromyogram provides a sensitive measure of neural respiratory drive with each breath and reflects the imposed load on respiratory muscles [36]. Similarly, the parasternal intercostal muscles are obligatory during respiration [37] and are the first external respiratory muscles to respond to an increased demand. Electrodes for diaphragm sEMG are typically placed in the 7th or 8th intercostal spaces along the midclavicular line, which is the zone of apposition where the active diaphragm thickens with consequent lung volume increase during breathing [38,39]. For the parasternal muscles, electrodes are typically placed in the 2nd or 3rd intercostal spaces along the midclavicular line. However, the historical summary of different locations has challenged the consistency and reliability of its sEMG signals, particularly with the interference of the large pectoralis major muscle [38]. This site for sEMG of parasternal has been studied through ultrasound imaging and it was found to be an area audible and visible for single motor unit activity with an acceptable signal-to-noise ratio [39].

The use of sEMG in critically ill patients undergoing invasive mechanical ventilation may provide a reliable assessment tool, however, it must be used with caution as the ventilatory modes can affect the observed results. For example, Roesthuis et al. [21], reported that the activity of the extra diaphragmatic inspiratory muscles increases when they remain at lower levels under support pressure. Furthermore, Cecchini et al. [27] showed that increasing levels of ventilatory support pressure and Neurally Adjusted Ventilatory Assist (NAVA) reduced the electrical activity of the diaphragm, scalene and alae nasal muscles. Nevertheless the study conducted by Bellani et al. [6] reported that electrical activity of the diaphragm, evaluated using sEMG, can be reliably used during ventilation with pressure support as it was analyzed and correlated with esophageal electrical measures. Additionally, Imsand et al. [31] study demonstrated that the duration of diaphragm and sternocleidomastoid electrical activities were similar with successive spontaneous and assisted breaths, except that the use of ventilation with support pressure has been preventing diaphragmatic fatigue [40].

Regarding patient positions during sEMG analysis, only the study conducted by Walterspacher et al. [26], revealed that the diaphragm muscle is the most active in supine and semi-sitting positions, while having reduced activation in the sitting position. The authors explained this phenomenon by attributing it to the compensation mechanism for changes in the length-tension of the diaphragm by the changes of body position, which unloads the diaphragm.

Systematic reviews of sEMG protocols in healthy individuals [11] adults and the elderly [12], were fundamental to research the evidence for sEMG in the ICU. A similar pattern was noticed with the immense variability of sEMG protocols and analyzes. In the context of the ICU, Abunurah et al. [5] performed a systematic review, not including details for processing sEMG or placing surface electrodes on the respiratory muscles, as described in the present systematic review.

During breathing in the upright position, other muscles besides the diaphragm are recruited, such as the scalenes and the parasternal intercostals. According to respiratory physiology, when high requirements for increased lung ventilation are present, recruitment of accessory respiratory muscles is required, characterized by the sternocleidomastoid and external intercostal muscles [11,41,42]. Although sEMG is an easy-to-apply and sensitive tool for capturing muscle activity, it has some disadvantages, such as susceptibility to interference from activities in other muscle groups (cross-talk) and limited standardization for analysis and processing of recorded sEMG signals [43].

This systematic review introduces some limitations that require careful consideration. The inconsistency of methods and data reports in the included studies restricted the possibility of conducting meta-analyses. For instance, some studies did not report any effect size measures with EMG signals, leading to an incomplete data assessment. Another fact is that despite the joint statement of ATS/ERS in 2002, which one of the goals was to promote standardization of techniques for assessing respiratory muscle function, including electromyographic methods, minimal adherence to this guideline was observed in the published studies.

Moreover, there is a lack of comparative evidence for different analysis techniques and removal strategies of motion artifacts, resulting in uncertainty regarding the best approach for reliable clinical inferences. Limited number of software is currently available to address the detection and removal of motion artifacts, and even manual inspection for noises has been reported. Once adequate signal processing is achieved, further research is needed to study the incorporation strategies of sEMG into clinical practices, including ICU.

## 5. Conclusion

The diaphragm and parasternal muscles are the most studied muscles in the cited protocols in the ICU setting, as well as their electrode placement patterns. There is significant variation in the methods and tools used for analyzing and processing of respiratory sEMG signals. The use of RMS appears to be essential in identifying muscle contractility behavior at different settings. Although clinical studies have provided useful information about the positions and analyses of respiratory sEMG in the ICU. Further studies are warranted to better explore the muscles physiological aspects in such a vulnerable population and tailor strategies to increase their chances of better outcomes.

## Supporting information

S1 Appendix Full search strategy. (DOCX)

S1 Table Results of the critical appraisal using the Newcastle-Ottawa scale adapted for cross-sectional studies. (DOCX)

S2 Table Results of the critical appraisal using the The Newcastle-Ottawa scale (NOS) for case-control study. (DOCX)

S3 Table Risk of bias assessment assessed by the Downs and Black. (DOCX)

S4 Table PRISMA checklist. (PDF)
