# Supplementary material for: Surface electromyography signal processing and evaluation on respiratory muscles of critically ill patients: A systematic review
Source: PLoS One. 2023 Apr 27;18(4):e0284911. doi: 10.1371/journal.pone.0284911 (PMC10138264; doi:10.1371/journal.pone.0284911)
Supplement: S1 Appendix — (DOCX) [file pone.0284911.s001.docx]

**S1 Appendix:** Full search strategy

Review Question:

Initial Logic grid aligned with the PECO elements of the Review Question

| **Population** | **Exposure** | **Comparator** | **Outcome measures** |
| --- | --- | --- | --- |
| Intensive care Units | Surface electromyography | none | Respiratory muscles |

Logic grid identified Keywords added (Pubmed/Medline: MesH and Entry terms)

| **Population** | **Exposure** | **Comparator** | **Outcome measures** |
| --- | --- | --- | --- |
| “**Intensive Care Units**”[Mesh] OR (Intensive Care Unit) OR (Unit, Intensive Care) OR (ICU, Intensive care units) OR “**Respiratory Care Units**”[Mesh] OR (Care Unit, Respiratory) OR (Care Units, Respiratory) OR (Respiratory Care Unit) OR (Unit, Respiratory Care) OR (Units, Respiratory Care) OR **“Critical Care”** [Mesh] OR (Care, Critical) OR (Intensive Care) OR (Care, Intensive) OR (Surgical Intensive Care) OR (Care, Surgical Intensive) OR (Intensive Care, Surgical) OR **“Critical illness” [mesh]** OR (critical illnesses) OR (illness, critical) OR (illnesses, critical) OR (critically ill). | **electromyography”** [Mesh] OR (Electromyographies) OR (Surface Electromyography) OR (Electromyographies, Surface) OR (Electromyography, Surface) OR (Surface electromyographies) OR (Electromyogram) OR (Electromyograms) | none | “**respiratory muscles”** [Mesh] OR (Muscle, Respiratory) OR (Muscles, Respiratory) OR (Respiratory Muscle) OR (Ventilatory Muscles) OR (Muscle, Ventilatory) OR (Muscles, Ventilatory) OR (Ventilatory Muscle) OR “**Diaphragm” [Mesh**] OR (Diaphragms) OR (Respiratory Diaphragm) OR (Diaphragm, Respiratory) OR (Diaphragms, Respiratory) OR (Respiratory Diaphragms) OR **“intercostal muscles” [Mesh]** OR (Intercostal Muscle) OR (Muscle, Intercostal) OR (Muscles, Intercostal). |

**Medical Literature Analysis and Retrievel System Online (Medline) via United States National Library of Medicine (PubMed)**

(((((((((((((((((((((((((((((((((((((((((((((((Intensive Care Units[Title/Abstract]) OR (Intensive Care Unit[Title/Abstract])) OR (Unit, Intensive Care[Title/Abstract])) OR (ICU Intensive Care Units[Title/Abstract])) OR (Respiratory Care Units[Title/Abstract])) OR (Care Unit, Respiratory[Title/Abstract])) OR (Care Units, Respiratory[Title/Abstract])) OR (Respiratory Care Unit[Title/Abstract])) OR (Unit, Respiratory Care[Title/Abstract])) OR (Units, Respiratory Care[Title/Abstract])) OR (Critical Care[Title/Abstract])) OR (Care, Critical[Title/Abstract])) OR (Intensive Care[Title/Abstract])) OR (Care, Intensive[Title/Abstract])) OR (Surgical Intensive Care[Title/Abstract])) OR (Care, Surgical Intensive[Title/Abstract])) OR (Intensive Care, Surgical[Title/Abstract])) OR (Critical illness[Title/Abstract])) OR (critical illnesses[Title/Abstract])) OR (illness, critical[Title/Abstract])) OR (illnesses, critical[Title/Abstract])) OR (critically ill[Title/Abstract])) AND (electromyography[Title/Abstract])) OR (Electromyographies[Title/Abstract])) OR (Surface Electromyography[Title/Abstract])) OR (Electromyographies, Surface[Title/Abstract])) OR (Electromyography, Surface[Title/Abstract])) OR (Surface electromyographies[Title/Abstract])) OR (Electromyogram[Title/Abstract])) OR (Electromyograms[Title/Abstract])) AND (respiratory muscles[Title/Abstract])) OR (Muscle, Respiratory[Title/Abstract])) OR (Muscles, Respiratory[Title/Abstract])) OR (Respiratory Muscle[Title/Abstract])) OR (Ventilatory Muscles[Title/Abstract])) OR (Muscle, Ventilatory[Title/Abstract])) OR (Muscles, Ventilatory[Title/Abstract])) OR (Ventilatory Muscle[Title/Abstract])) OR (Diaphragm[Title/Abstract])) OR (Diaphragms[Title/Abstract])) OR (Respiratory Diaphragm[Title/Abstract])) OR (Diaphragm, Respiratory[Title/Abstract])) OR (Diaphragms, Respiratory[Title/Abstract])) OR (Respiratory Diaphragms[Title/Abstract])) OR (intercostal muscles[Title/Abstract])) OR (Intercostal Muscle[Title/Abstract])) OR (Muscle, Intercostal[Title/Abstract])) OR (Muscles, Intercostal[Title/Abstract])

**WEB OF SCIENCE**

((TS=(Intensive Care Units OR Intensive Care Unit OR Unit, Intensive Care OR ICU, Intensive care units OR Respiratory Care Units OR Care Unit, Respiratory OR Care Units, Respiratory OR Respiratory Care Unit OR Unit, Respiratory Care OR Units, Respiratory Care OR Critical Care OR Care, Critical OR Intensive Care OR Care, Intensive OR Surgical Intensive Care OR Care, Surgical Intensive OR Intensive Care, Surgical OR Critical illness OR critical illnesses OR illness, critical OR illnesses, critical OR critically ill)) AND TS=(electromyography OR Electromyographies OR Surface Electromyography OR Electromyographies, Surface OR Electromyography, Surface OR Surface electromyographies OR Electromyogram OR Electromyograms)) AND TS=(respiratory muscles OR Muscle, Respiratory OR Muscles, Respiratory OR Respiratory Muscle OR Ventilatory Muscles OR Muscle, Ventilatory OR Muscles, Ventilatory OR Ventilatory Muscle OR Diaphragm OR Diaphragms OR Respiratory Diaphragm OR Diaphragm, Respiratory OR Diaphragms, Respiratory OR Respiratory Diaphragms OR intercostal muscles OR Intercostal Muscle OR Muscle, Intercostal OR Muscles, Intercostal)

**SCIENCE DIRECT**

(“Intensive Care Unit” OR “Critical Care” OR “Intensive Care” OR “critically ill”) AND (electromyography OR “Surface Electromyography”) AND (“respiratory muscles” OR “Diaphragm” OR “intercostal muscles”)

**SCOPUS**

TITLE-ABS-KEY("intensive care" OR "critical care" OR "critical illness" OR "ICU" OR "respiratory care unit" OR "critically ill" OR "surgical intensive care" AND electromyography OR electromyogram AND "respiratory muscles" OR "ventilatory muscles" OR "diaphragm" OR "intercostal muscles")

**Cumulative Index to Nursing and Allied Health Literature (CINAHL)**

intensive care unit OR icu OR critical care OR critical care unit AND electromyography AND respiratory muscles
