# Supplementary material for: Surface electromyography signal processing and evaluation on respiratory muscles of critically ill patients: A systematic review
Source: PLoS One. 2023 Apr 27;18(4):e0284911. doi: 10.1371/journal.pone.0284911 (PMC10138264; doi:10.1371/journal.pone.0284911)
Supplement: S1 Table — (DOCX) [file pone.0284911.s002.docx]

**S2 Table.** Results of the critical appraisal using the Newcastle-Ottawa Scale (NOS) adapted for cross-sectional studies.

The Newcastle-Ottawa Scale (NOS) scale was clearly evaluated for distinction, comparability and exposure, receiving scores from zero to nine, case-control and cross-sectional studies were scored separately, classifying as: Good quality: 7-9 points; Satisfactory quality: 5-6 points; Poor quality: 0-4 points.

| Study | Study  Design | Selection | | | | Comparability | Outcome | | |
| --- | --- | --- | --- | --- | --- | --- | --- | --- | --- |
|  |  | Representative sample? | Sample size | Non-respondents | Ascertainment of the exposure | Based on Design or Analysis | Assessment of outcome | Statistical test | Total Score |
| Pozzi, 2022 [17] | Cross-sectional | - | - | + | + + | - | ++ | + | 6 |
| Graßhoff, 2021 [18] | Cross-sectional | - | - | + | + + | - | + + | + | 6 |
| Bureau, 2021 [19] | Cross-sectional | - | + | + | + + | - | + + | + | 7 |
| Lokin, 2020 [20] | Cross-sectional | - | + | + | + + | - | + + | + | 7 |
| Roesthuis, 2020 [21] | Cross-sectional | - | + | + | + + | - | + + | + | 7 |
| Souza costa, 2020 [22] | Cross-sectional | - | + | + | + + | - | + + | + | 7 |
| Bellani, 2018 [6] | Cross-sectional | - | - | - | + + | - | + + | + | 5 |
| Duarte, 2017 [23] | Cross-sectional | - | - | - | + + | - | + + | + | 5 |
| Ortega, 2017 [25] | Cross-sectional | - | + | - | + + | - | + + | + | 6 |
| Walterspacher, 2017 [26] | Cross-sectional | - | - | + | + + | - | + + | + | 6 |
| Cecchini, 2014 [27] | Cross-sectional | - | + | - | + + | - | + + | + | 6 |
| Schmidt, 2013 [28] | Cross-sectional | - | - | - | + + | - | + + | + | 5 |
| Tassaux, 2005 [29] | Cross-sectional | - | - | - | + + | - | + + | + | 5 |
| Tassaux, 2002 [30] | Cross-sectional | - | - | + | + + | - | + + | + | 6 |
| Imsand, 1994 [31] | Cross-sectional | - | - | - | + + | - | + + | + | 5 |
