# Supplementary material for: Surface electromyography signal processing and evaluation on respiratory muscles of critically ill patients: A systematic review
Source: PLoS One. 2023 Apr 27;18(4):e0284911. doi: 10.1371/journal.pone.0284911 (PMC10138264; doi:10.1371/journal.pone.0284911)
Supplement: S2 Table — (DOCX) [file pone.0284911.s003.docx]

**S3 Table.** Results of the critical appraisal using the The Newcastle-Ottawa Scale (NOS) for case-control study.

| Study | Study  Design | Selection | | | | Comparability | Outcome | | |  |
| --- | --- | --- | --- | --- | --- | --- | --- | --- | --- | --- |
|  |  | Case Definition Adequate? | Case Representative? | Selection  of Controls | Definition of Controls | Based on Design or Analysis | Ascertainment of  exposure | Same Method for Case and Control | Non-Response rate | Total Score |
| Sánchez*,* 2017 [24] | Case-control | + | + | - | + | + + | + | + | - | 7 |
